# Ultrasonographic Assessment of the Thymus Among Neonates Admitted in a Special Newborn Care Unit of a Medical College Hospital in Eastern India

**DOI:** 10.7759/cureus.67036

**Published:** 2024-08-16

**Authors:** Saumyen DE, Sandip K Mandal, Rashmita Das, Debabrata Maitra, Sreetama Saha, Rishana Shahin K S, Debangshi Ghosh, Anwesha Dey

**Affiliations:** 1 Paediatrics, College of Medicine & Sagore Dutta Hospital, Kolkata, IND; 2 Radiodiagnosis, College of Medicine & Sagore Dutta Hospital, Kolkata, IND

**Keywords:** special neonatal care unit, thymic ultrasonography, birth weight, neonate, thymic index

## Abstract

Introduction: The thymus is essential for the maturation of T-lymphocytes, crucial for adaptive immunity. In neonates, thymic development is influenced by factors such as gestational age, birth weight, birth length, etc. Ultrasonography offers a non-invasive method to assess thymic size and morphology. However, there is limited research on thymic ultrasonography among neonates in Eastern India. This study aimed to investigate the ultra-sonographic characteristics of the thymus in neonates admitted to a Special Neonatal Care Unit (SNCU) in Eastern India and its correlation with certain clinical and anthropometric variables.

Materials and methods: Conducted from May to July 2024, this cross-sectional observational study involved 80 neonates admitted to the SNCU at the College of Medicine & Sagore Dutta Hospital. Thymic ultrasonography, using an Esaote MylabX7 ultrasound machine with a 3-11 MHz linear probe (Esaote, Genoa, Italy), measured thymic length, width, anteroposterior (AP) dimension, sagittal area, and thymic index. Clinical data, including gestational age, birth weight, birth length, and antibiotics received, were collected from medical records. Pearson's correlation coefficient and linear regression analysis were used to examine correlations and predictors of thymic dimensions.

Results: The cohort consisted of 45 boys (56%) and 35 girls (44%). The average birth weight was 2,626 grams for boys and 2,639 grams for girls. The median gestation period was 38 weeks for both groups. Thymic measurements did not significantly differ by gender. Correlation analysis revealed significant relationships between thymic dimensions and neonatal anthropometric measurements. Birth weight showed a strong positive correlation with thymic length (r = 0.486) and width (r = 0.233). Linear regression identified birth weight as a significant predictor of the thymic index (p < 0.001), explaining 21.2% of the variance.

Conclusion: This study provides insights into the factors associated with thymic size in neonates, highlighting the critical role of birth weight in thymic development. Future research should explore additional variables influencing thymic size and consider larger sample sizes to enhance model explanatory power and its potential role in immunity.

## Introduction

The thymus, a primary lymphoid organ, provides a crucial microenvironment for the proliferation, differentiation, and selection of T-lymphocytes, which are key players in cellular immunity [1]. T-cell differentiation is a highly orchestrated process. Mature, immunocompetent T cells leave the thymus via blood and lymphatic vessels and migrate to peripheral lymphoid organs, such as the spleen and lymph nodes. These T-cells are essential for adaptive immunity [2-7], and thereby thymus plays an important role in immune development. In neonates, the thymus undergoes rapid growth [8,9] and subsequently involutes during the later part of infancy [1,10].

Thymus atrophy is associated with severe malnutrition and increased morbidity and proneness to neonatal infections [3,11]. Thymus size at birth is a significant indicator of immune competence [12]. Various factors, including gestational age, birth weight, and length, can influence thymic development [1,2,6,7,13].

A crude estimate of the thymus can be done by chest radiography, but it involves radiation exposure, and only transverse diameter can be ascertained by this. Since the thymus gland varies widely in size and shape in infants, the estimation of thymus size based on X-ray is not reliable [14]. Ultrasonography is a non-invasive and reliable method for assessing thymic size and morphology in neonates [5,13,15].

Despite the importance of thymic function in neonatal immunity, limited research has been conducted on thymic ultrasonography among neonates in Eastern India. In this study, we aimed to investigate the ultrasonographic characteristics of the thymus among neonates admitted to a Special Neonatal Care Unit (SNCU) of a tertiary care hospital in Eastern India and its correlation with certain clinical and anthropometric variables such as birth weight, gender, gestation weeks, birth length, and antibiotics received during hospital stay.

## Materials and methods

Study design, setting, and participants

This cross-sectional observational study was conducted at the SNCU of College of Medicine & Sagore Dutta Hospital, a tertiary care hospital in Eastern India, from May to July 2024 after obtaining approval from the Institutional Ethics Committee (IEC), College of Medicine & Sagore Dutta Hospital, as indicated by the clearance certificate bearing memo number CMSDH/IEC/53/05-2024, dated 13th May 2024. The study was registered under IEC (Regn No: ECR/1210/Inst/WB/2019/RR-22).

The study population included all neonates admitted to the SNCU during the study period. At first, 85 neonates were enrolled using a census method. Among those babies, 80 newborns were finally included after obtaining consent from parents/guardians. Five babies were excluded based on exclusion criteria - three babies were abandoned (whose birth details such as date of birth, period of gestation, and birth weight were not available) and two babies had gross birth defects.

Data collection

**Figure 1:**
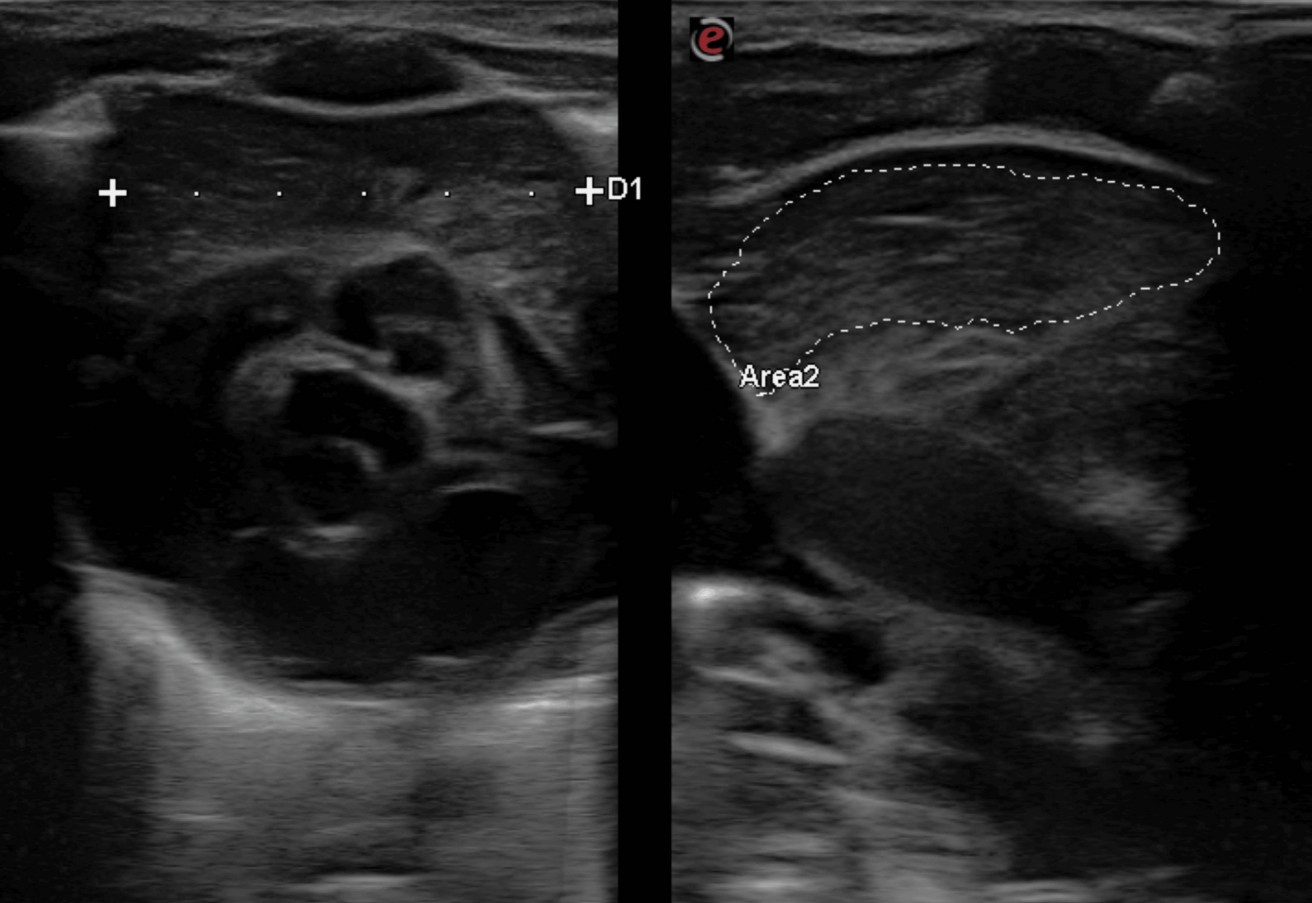
Ultrasonographic image of the thymus showing transverse diameter (D1) and sagittal area (Area2)

Thymic ultrasonography was performed using the Esaote MylabX7 ultrasound machine with a 3-11 MHz linear probe (Esaote, Genoa, Italy). Thymic measurements, including length (cephalocaudal dimension on sagittal plane), width (maximum dimension on transverse plane, as shown in Figure 1), anteroposterior (AP) dimension, sagittal area (maximum area on sagittal plane, as shown in Figure 1), and thymic index (width × sagittal area) [15], were recorded using a trans-sternal approach.

Clinical data, such as gestational age, birth weight, birth length, and antibiotics received during hospital stay, were collected from medical records.

Statistical analysis

Statistical analysis was performed using the software Jamovi 2.4.8 (https://www.jamovi.org/download.html). It examined the correlations and associations between thymic measurements and clinical parameters. Pearson's correlation coefficient was used to determine the strength and direction of linear relationships. Linear regression analysis was performed to investigate predictors of thymic dimensions. A p-value <0.05 was considered statistically significant.

## Results

The study cohort consisted of 80 newborns, with 45 boys (56%) and 35 girls (44%). The average birth weight for boys was 2,626 grams (SD = 554 g), while for girls, it was 2,639 grams (SD = 511 g). Both groups had a median gestation period of 38 weeks, with an interquartile range (IQR) of two weeks for both boys and girls. The mean age for boys and girls was 5.42 days and 5.77 days, respectively.

Table 1 provides a comparative view of thymic measurements between boys and girls, highlighting any differences and variability within each group. The difference in various thymic measurements based on gender is not statistically significant.

**Table 1:** Comparison of different thymic measurements based on gender SD = standard deviation, Statistical test * - Independent *t*-test used where the dataset followed a normal distribution, and the Mann-Whitney U test was applied elsewhere. mm = millimeter, AP = antero-posterior dimension

Measurement	Boys (Mean ±SD)	Girls (Mean ± SD)	*p*-value ( Statistical Test *)
Thymic Length (mm)	28.2 ± 4.54	27.3 ± 4.28	0.58 (Mann-Whitney U test)
Thymic Width (mm)	19.7 ± 4.33	20.7 ± 4.38	0.17 (Mann-Whitney U test)
Thymic AP (mm)	7.60 ± 2.07	7.51 ± 1.74	0.84 (Independent *t*-test)
Sagittal Area (cm²)	2.41 ± 0.961	2.40 ± 0.818	0.98 (Independent *t*-test)
Thymic Index (cm³)	4.91 ± 2.78	5.10 ± 2.29	0.48 (Mann-Whitney U test)

In our study, we analyzed the relationships between various anthropometric parameters and clinical variables related to thymic dimensions and neonatal characteristics. The variables included thymic length (mm), thymic width (mm), sagittal area (cm²), thymic index (width × sagittal area) (cm³), birth weight (gm), birth length (cm), gender, and antibiotics received or not. We used Pearson's correlation coefficient (r) to determine the strength and direction of the linear relationships between these variables. The results are summarized in Table 2.

**Table 2:** Correlation matrix of thymic measurements and clinical variables among neonates df = degrees of freedom, mm = millimeter, cm = centimeter, cm^2^ = square centimeter

Variables	Pearson's r	df	*p*-value
Thymic length (mm) vs Thymic Width (mm)	0.456	78	< 0.001
Thymic length (mm) vs Sagittal AREA (cm²)	0.712	78	< 0.001
Thymic length (mm) vs Thymic index (cm³)	0.718	78	< 0.001
Thymic length (mm) vs BIRTH WEIGHT (gm)	0.486	78	< 0.001
Thymic length (mm) vs Birth Length (cm)	0.407	78	< 0.001
Thymic length (mm) vs GENDER	-0.105	78	0.352
Thymic length (mm) vs ANTIBIOTICS RECEIVED	0.2	78	0.075
Thymic Width (mm) vs Sagittal AREA (cm²)	0.387	78	< 0.001
Thymic Width (mm) vs Thymic index (cm³)	0.73	78	< 0.001
Thymic Width (mm) vs BIRTH WEIGHT (gm)	0.233	78	0.038
Thymic Width (mm) vs Birth Length (cm)	0.065	78	0.569
Thymic Width (mm) vs GENDER	0.121	78	0.283
Thymic Width (mm) vs ANTIBIOTICS RECEIVED	-0.181	78	0.107
Sagittal AREA (cm²) vs Thymic index (cm³)	0.893	78	< 0.001
Sagittal AREA (cm²) vs BIRTH WEIGHT (gm)	0.453	78	< 0.001
Sagittal AREA (cm²) vs Birth Length (cm)	0.242	78	0.03
Sagittal AREA (cm²) vs GENDER	-0.003	78	0.978
Sagittal AREA (cm²) vs ANTIBIOTICS RECEIVED	0.145	78	0.199
Thymic index (cm³) vs BIRTH WEIGHT (gm)	0.415	78	< 0.001
Thymic index (cm³) vs Birth Length (cm)	0.195	78	0.083
Thymic index (cm³) vs GENDER	0.036	78	0.748
Thymic index (cm³) vs ANTIBIOTICS RECEIVED	-0.002	78	0.985
BIRTH WEIGHT (gm) vs Birth Length (cm)	0.717	78	< 0.001
BIRTH WEIGHT (gm) vs GENDER	0.012	78	0.917
BIRTH WEIGHT (gm) vs ANTIBIOTICS RECEIVED	0.174	78	0.124
Birth Length (cm) vs GENDER	-0.07	78	0.534
Birth Length (cm) vs ANTIBIOTICS RECEIVED	0.354	78	0.001
GENDER vs ANTIBIOTICS RECEIVED	0.074	78	0.514

The correlation matrix highlights several significant relationships between thymic dimensions and neonatal anthropometric measurements. The strong correlations between thymus length, thymic width, sagittal area, and thymic index suggest that these variables are closely related in their contributions to thymic size. Birth weight and length are also important factors associated with thymic measurements. However, gender and antibiotics received do not show significant correlations with thymic dimensions, indicating other factors may play a more critical role in these associations.

The size of the thymus was assessed by ultrasonography to estimate the volume, referred to as the thymic index, which closely correlates with the actual volume [12,15]. Owing to a skewed distribution of the thymic index, a logarithmic transformation of the thymic index was used. Then, linear regression analysis was done to explore the predictors of the thymic index (width x sagittal area) in newborns. The independent variables included birth weight, gender, length, and gestational age. The model fit and specific results are summarized in Table 3.

**Table 3:** Geometric mean ratios for log-transformed thymic index: a linear regression analysis SE = standard error of the estimate

Predictor	Estimate	GMR	95% CI for GMR	SE	*t*-value	*p*-value
Intercept	1.11593	3.052	(0.819, 11.39)	0.7177	1.555	0.124
Birth Weight (gm)	2.44e−4	1.000244	(1.000118, 1.000370)	6.62e−5	3.689	< 0.001
Birth Length (cm)	-0.02085	0.979	(0.949, 1.009)	0.016	-1.304	0.196
Gestation (wks)	-0.00316	0.997	(0.968, 1.027)	0.0151	-0.209	0.835
Gender (Boy=1, Girl=2)	0.02147	1.022	(0.935, 1.118)	0.0453	0.474	0.637

This linear regression model identified birth weight as a significant predictor of the thymic index in newborns, with an R² value of 0.212. The model showed a moderate positive correlation (R = 0.46) between observed and predicted values. Birth weight is the only statistically significant predictor (p < 0.001), with a GMR of 1.000244, indicating that each additional gram of birth weight is associated with a 0.0244% increase in the geometric mean of the thymic index. However, gender, length, and gestational age were not significant predictors as per this model.

## Discussion

In our study, we investigated the differences and similarities in thymic measurements between boys and girls, the correlations between various neonatal characteristics and thymic dimensions, and the predictors of thymic index in newborns. Our analysis revealed no statistically significant differences in thymic measurements between boys and girls. The mean thymic length, width, AP dimension, sagittal area, and thymic index were comparable between genders, with p-values well above the threshold for significance (p > 0.05). This finding aligns with previous research, such as Hasselbalch et al. [2], which also reported no significant gender differences in thymic size among healthy neonates. Another study done by Magu et al. [5] also found that there is no statistically significant difference in thymic index based on the gender of the child. However, in a study done by Liang et al. [16], the mean thymus size was larger among boys than girls.

The correlation analysis highlighted several significant relationships between thymic dimensions and neonatal anthropometric measurements. Thymic length and width were significantly correlated with birth weight (r = 0.486 and r = 0.233, respectively), indicating that larger birth weight is associated with larger thymic dimensions. This is consistent with the findings of Eriksen et al. [9], Iscan et al. [14], Aaby et al. [12], and Jeppesen et al. [13] who also reported a positive association between birth weight and thymic size at birth among both low and normal birth weight infants. However, this finding is not in alignment with the result obtained by the study done by Magu et al. [5], who demonstrated that thymus volume does not have any statistically significant corelation with birth weight.

In our study, we did not find any statistically significant corelation of thymic index with birth length, which echoes the findings of study done by Hasselbalch et al. [2]. We aimed to identify predictors of the thymic index by linear regression analysis. The model for log transformed thymic index identified birth weight as a significant predictor (p < 0.001), while gender, length, and gestational age were not statistically significant. The model explained 21.2% of the variance in thymic index, indicating that birth weight plays a crucial role in determining thymic dimensions. These findings are in line with previous studies, done by Varga et al. [1], Hasselbalch et al. [2], and Eriksen et al. [9], who also reported birth weight as a significant predictor of thymic size. The modest R² values in our models suggest that other factors not included in the analysis may also influence thymic size, warranting further research to explore additional variables.

Strengths of our study: Ultrasound measurements were performed by one experienced examiner, thereby eliminating inter-observer variations.

Drawback: This is a single-center study and has a smaller sample size.

## Conclusions

Our study provides valuable insights into the factors associated with thymic size in neonates. The lack of significant gender differences and the strong correlations between thymic dimensions highlight the interrelated nature of these measurements. Birth weight emerged as a critical predictor of thymic size, underscoring its importance in neonatal thymic development. Future research should aim to identify other potential factors influencing thymic size and consider larger sample sizes to enhance the explanatory power of the models and their potential role in immunity.
